# Electrostatic Charge Effects on Pharmaceutical Aerosol Deposition in Human Nasal–Laryngeal Airways

**DOI:** 10.3390/pharmaceutics6010026

**Published:** 2014-01-29

**Authors:** Jinxiang Xi, Xiuhua Si, Worth Longest

**Affiliations:** 1 Department of Mechanical and Biomedical Engineering, Central Michigan University, Mt Pleasant, MI 48858, USA; 2 Department of Engineering, Calvin College, Grand Rapids, MI 49546, USA; E-Mail: xs22@calvin.edu; 3 Department of Mechanical and Nuclear Engineering, Virginia Commonwealth University, Richmond, VA 23284, USA; E-Mail: pwlongest@vcu.edu

**Keywords:** nasal drug delivery, charged particles, image-based modeling, nasal deposition

## Abstract

Electrostatic charging occurs in most aerosol generation processes and can significantly influence subsequent particle deposition rates and patterns in the respiratory tract through the image and space forces. The behavior of inhaled aerosols with charge is expected to be most affected in the upper airways, where particles come in close proximity to the narrow turbinate surface, and before charge dissipation occurs as a result of high humidity. The objective of this study was to quantitatively evaluate the deposition of charged aerosols in an MRI-based nasal–laryngeal airway model. Particle sizes of 5 nm–30 µm and charge levels ranging from neutralized to ten times the saturation limit were considered. A well-validated low Reynolds number (LRN) *k–ω* turbulence model and a discrete Lagrangian tracking approach that accounted for electrostatic image force were employed to simulate the nasal airflow and aerosol dynamics. For ultrafine aerosols, electrostatic charge was observed to exert a discernible but insignificant effect. In contrast, remarkably enhanced depositions were observed for micrometer particles with charge, which could be one order of magnitude larger than no-charge depositions. The deposition hot spots shifted towards the anterior part of the upper airway as the charge level increased. Results of this study have important implications for evaluating nasal drug delivery devices and for assessing doses received from pollutants, which often carry a certain level of electric charges.

## 1. Introduction

Electrostatic charges are introduced via induction or conduction in most aerosol generation and transport processes. Aerosol droplets generated by atomization are often highly charged [1]. Pharmaceutical powders are composed of fine particles in contact with each other or with device walls, which can acquire charges via electron exchange due to different surface potentials [2]. The electric charges carried by individual particles play an important role in the behavior and fate of inhaled pharmaceutical aerosols. Such electrostatic effects are expected to be significant in the upper airways before these electric charges dissipate due to high humidity. Furthermore, the nasal passages are narrow. Charge effects may be even more pronounced in the nasal airways where particles come in close proximity to large surfaces areas.

A number of theoretical and experimental studies have considered the charge effects on the deposition of respiratory aerosols [2,3,4,5,6,7,8]. The deposition of pharmaceutical particles in the nose is influenced by different mechanisms. The main mechanisms are impaction, sedimentation, Brownian diffusion, and electrostatic deposition [9]. By gradually reducing the particle charges in a nasal replica cast, Fry [6] reported that charged, partially neutralized, and Boltzmann charged particles had similar deposition characteristics in the nasal cavity. However, the charge levels in the above study are well below those of typical metered dose inhalers (MDI) that are sufficiently high in order to alter deposition of the inhaled particles in the respiratory tract [7]. Boltzmann charge on submicron aerosols can be significant [10] and may cause some level of deposition that was not identified by Fry [6]. Considerable deposition enhancements have been reported in the larynx and tracheobronchial casts for both micrometer [3] and submicron [5] charged particles. Aerosols from a dry powder inhaler (DPI) can also acquire a high level of charge, the impact of which has been recently reviewed by Karner and Urbanetz [2]. Yu and coworkers [8,11] theoretically studied the electrostatic precipitation in duct flows and proposed empirical correlations for charged aerosol depositions in such idealized geometries. However, these correlations have been based on fully developed flows and cannot be readily extended to complex geometries such as the respiratory tract where developing flow prevails. The subsequent questions are: how well can these expressions determine deposition rates in the nasal cavity? Or, how can the existing nasal correlations as proposed in Xi *et al.* [12] be extended to include particle charge effects? Even more interestingly, how can the nasal drug delivery be improved by taking advantage of the electrostatic charges present in the particles?

As a pilot study to answer the above questions, the objective of this paper was to quantitatively evaluate the effect of charge on the deposition of pharmaceutical aerosols in an adult nasal–laryngeal airway model during inhalation. This entailed three specific aims: (1) validating the electrostatic model in a simple duct geometry; (2) evaluating the influence of charge levels on micron and submicron particles in the nasal–laryngeal airway; and (3) quantifying the total and sub-regional depositions typical of MDI and DPI aerosols. Results of this study will have important implications in evaluating nasal drug delivery devices and in assessing health risks from exposure to environmental pollutants, which often carry a certain level of electrostatic charges.

## 2. Methods

### 2.1. Nasal–Laryngeal Airway Model

**Figure 1:**
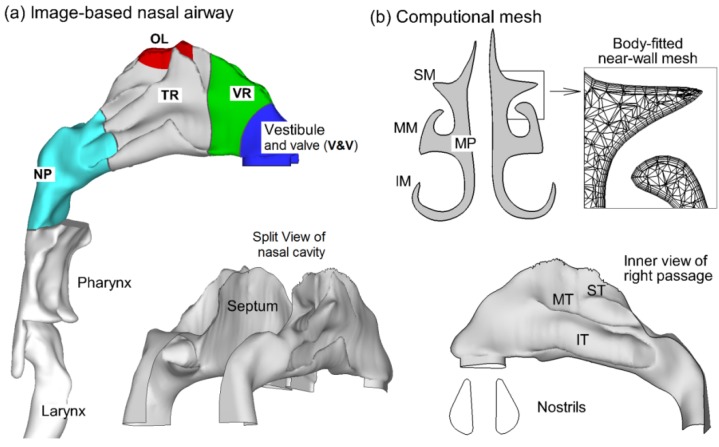
(**a**) Surface geometry and inner anatomy of the nasal airway of an adult; (**b**) Computational mesh. SM, superior meatus; MM, middle meatus; IM, inferior meatus; MP, median passage; ST, superior turbinate; MT, middle turbinate; IT, inferior turbinate.

A physiologically accurate airway model is necessary for reliable analysis of inhalation medication dosing. Image-based modeling represents a remarkable improvement over conventional cadaver casting that is subject to large distortions due to the shrinkage of mucous membranes or insertion of casting materials. To construct the airway model, MRI scan tracings of the nasal cavity of a healthy non-smoking 53-year-old male (weight 73 kg, height 173 cm) were used in this study. The multi-slice tracings were first segmented into 3-D model using MIMICS (Materialise, Ann Arbor, MI, USA). This 3-D model was further converted to a set of contours to define the airway of interest. Based on these contours, an internal surface geometry was constructed in Gambit (Ansys, Inc., Canonsburg, PA, USA), as shown in Figure 1a. Detailed morphometric information about coronal cross-sectional area, perimeter, and hydraulic diameters for the right and left passages could be found in Xi and Longest [12]. The resulting model was intended to faithfully represent the anatomy of the nasal airway with only minor surface smoothing. This model could either be manufactured into a solid cast by prototyping techniques for *in vitro* studies, or be meshed with high-quality computational elements for numerical analysis. In Figure 1a, the nasal septum divided the nose into right and left passages; the nasal conchae (or turbinates) further separated each nasal passage into three layers, namely the inferior meatus (IM), middle meatus (MM), and superior meatus (SM). Figure 1b showed the computational mesh of the airway model that was generated with ANSYS ICEM 12 (Ansys, Inc., Canonsburg, PA, USA). Considering the high complexity of the model geometry, an unstructured tetrahedral mesh was created with high-resolution prismatic elements in the near-wall region.

### 2.2. Fluid-Particle Electrostatic Model

Flows in this study were assumed to be isothermal and incompressible. Steady inhalation was assumed for all simulations. Normal breathing condition (20 L/min) for an adult was simulated. The mean inlet Reynolds number at the nostrils was about 2234. The maximum Reynolds number based on the hydraulic diameter of the glottal aperture was approximately 5000. The low Reynolds number (LRN) *k–ω* model was selected based on its ability to accurately predict multi-regime flows.

Particles with diameters (*d*_p_) ranging from 5 nm to 30 µm were considered in this study. Typical pharmaceutical particles are in micrometer range. Nanoparticles were considered due to recent interests in nanomedicines for direct-nose-to-brain drug delivery [13]. Nasal spray droplets can be larger than 30 µm. The charge effect on such droplets was discussed later in Results, Section 3.3. Particle trajectories were calculated on a Lagrangian basis by directly integrating an appropriate form of the particle transport equation. Aerosols of this size range (5 nm–30 µm) had very low Stokes numbers (*St*_k_ = ρ_p_*d*_p_^2^*UC*_c_*/18*μ*D*_h_ << 1), with ρ_p_ being the particle density (1.0 g/cm^3^), *C*_c_ the Cunningham slip correction factor, μ the fluid viscosity, *U* the mean fluid velocity, and *D*_h_ the hydraulic diameter of the nostril. The governing equation for spherical particle motion under these conditions could be expressed as

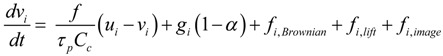
(1)
with *v_i_* being the particle velocity, *u_i_* the local fluid velocity, and τ*_p_* (*i.e.*, ρ_p_*d*_p_^2^/18µ*)* the characteristic time required for a particle to respond to changes in the flow field. The drag factor *f*, which represented the ratio of the drag coefficient *C*_D_ to Stokes drag, was based on the expression of Morsi and Alexander [14] for aerosols greater than 1 µm. It also included the Cunningham slip correction factor for sizes less than 1 µm. The force per unit particle mass due to gravity was included with the gravity vector oriented in the vertical direction. Saffman lift was calculated for particles greater than 1 µm. The effect of Brownian motion was considered for particles less than 1 µm [15,16]. A charged particle near a conducting solid would experience an attractive image force [10]. For a particle with a charge *q* in Coulombs, the image force per unit mass could be expressed as

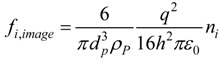
(2)
with *h* being the height of the particle above the conducting surface and ε_0_ (=8.854 × 10^−12^ C^2^/Nm^2^) being the permittivity of free space. The unit vector *n_i_* is normal to the surface and points away from the space occupied by the air passage, such that the image force always acts in the direction of the wall. Based on the assumption of a dilute aerosol, no particle-to-particle electrostatic interactions were modeled, *i.e.*, the space charge effect was not considered.

### 2.3. Charge Level in Pharmaceutical Aerosols

A number of studies have measured the charge level on standard pharmaceutical aerosols from DPI and MDI devices [7,17]. Generally, the charge on pharmaceutical aerosols depends on both the size and physical properties of such aerosols. The larger a particle, the more charge it can hold. Moreover, the charge on MDI aerosols is about one order of magnitude greater than that on DPI aerosols. Vinchurkar *et al.* [18] showed that the saturation charge limit was a reasonable first order approximation for the charge on DPI aerosols. As a result, these first order approximations were used in this study to define the size-dependent charge characteristics of DPI (saturation limit) and MDI (ten times the saturation limit) aerosols.

### 2.4. Numerical Method and Grid Sensitivity Analysis

Fluent (Ansys, Inc., Canonsburg, PA, USA) was employed to solve the governing mass and momentum conservation equations. User-supplied Fortran and C programs were implemented for the calculation of initial particle profiles, wall mass flow rates, Brownian force [19], image force [18], and near-wall velocity interpolation [12,20]. All transport equations were discretized to at least second order in space. Convergence of the flow field solution was assumed when the normalized global mass residuals fell below 10^−5^ and the residual-iteration curves for all flow parameters became asymptotic. The computational mesh of the nasal airway geometry was generated with ANSYS ICEM. A grid sensitivity analysis was conducted by testing the effects of different mesh densities with approximately 0.6 million, 0.96 million, 1.5 million, 2.4 million elements. The predicted deposition rate was less than 1% when increasing mesh size from 1.5 million to 2.4 million. As a result, the final grid for reporting flow field and deposition conditions consisted of approximately 1.5 million cells and had a constant near-wall cell height of 0.05 mm.

## 3. Results and Discussion

### 3.1. Validation of Electrostatic Deposition Model

**Figure 2:**
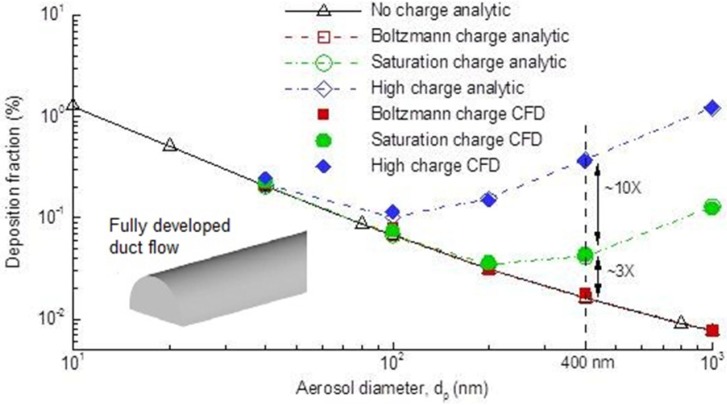
Comparison of numerically predicted aerosol deposition due to the image force with the analytic expression of Chen and Yu [11] for fully developed laminar flow in a tube.

To ensure that the electrostatic image force was correctly modeled, predicted deposition in a duct was compared with the analytical expression of Chen and Yu [11]. Characteristics of the duct flow system were a diameter of 0.45 cm, a length of 5.6 cm, and a Reynolds number of 556, resulting in laminar flow conditions. Particles ranging from 1 nm to 1 µm were considered with three charge levels (*i.e.*, Boltzmann, saturation, and high charge). A good agreement was obtained between the computational fluid dynamics (CFD) predictions and theoretical results of Chen and Yu [11] for all three of the charges considered, as shown in Figure 2, indicating that the CFD approach hereof adequately captured the deposition of charged aerosols. The depositions with no charge and with Boltzmann charge were observed to fall into the same curve, indicating negligible influence from Boltzmann charge. For higher charge levels, the electrostatic effect enhanced the tubular deposition and this effect amplified with increasing particle size. Specifically, for 400 nm particles, the deposition fraction with high charge and saturation charge was about 30 and 3 times than that with no charge, respectively.

### 3.2. Nasal Airflows

**Figure 3:**
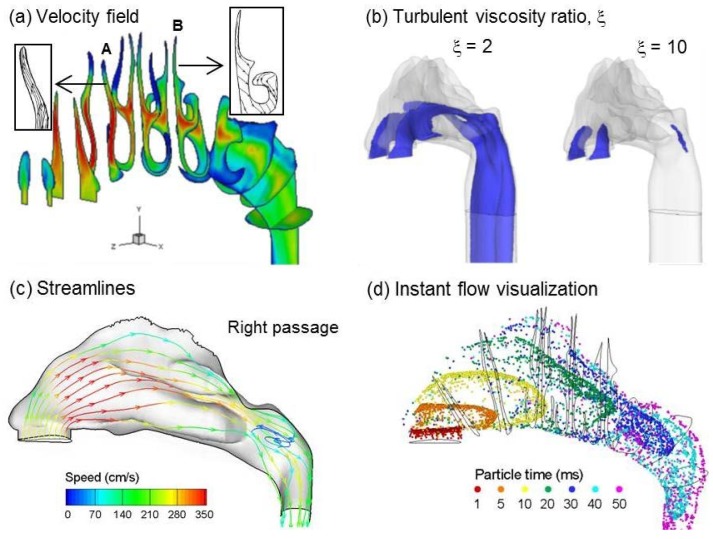
Inhalation airflow inside the nasal airway under normal breathing conditions: (**a**) velocity field; (**b**) turbulent viscosity ratio; (**c**) streamlines (right passage); and (**d**) flow pattern visualized with mass-less fluid particles at various instants.

Steady state flow fields in the nasal airway for an inhalation flow rate of 20 L/min were shown in Figure 3. Airflow in the nasal cavity was mostly laminar or transitional under this breathing condition, with turbulence occurring mainly in the nasal vestibule-valve region and dorsal nasopharynx (Figure 3b). Laminar flow dominated in the main nasal passage. A recirculation zone was observed in the upper dorsal part of the nasopharynx as a result of sudden area expansion in both cross-sectional and effective (*i.e.*, hydraulic) flow areas (Figure 3c). The magnitude of the secondary velocity in each slice was approximately 30% of the main flow. This secondary motion functioned to distribute the inhaled air into each fin-like meatus. Because of the dramatic airway bend from the nostrils to the nasopharynx, the aerosol front constantly adjusted its direction by following the mean streamline curvature of inhaled airflows (Figure 3d). Faster transport and deeper penetration of aerosols were apparent in the medial passages while slow-moving particles were found near the airway walls.

### 3.3. Charged Particle Depositions

**Figure 4:**
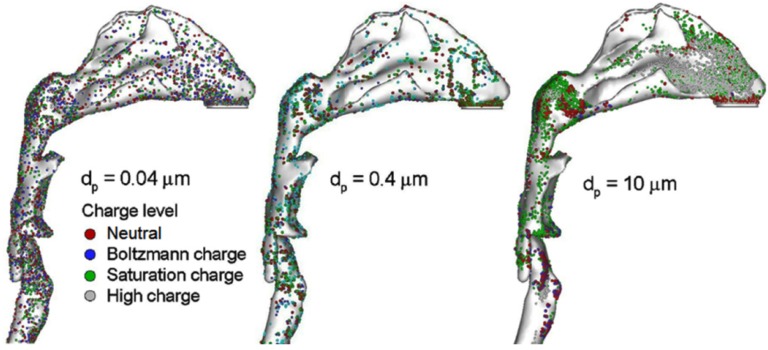
Surface deposition of monodisperse aerosols with different charge levels and particle sizes.

Figure 4 showed the comparison of deposition pattern among ultrafine (0.04 µm), fine (0.4 µm) and coarse (10 µm) aerosols under different charge levels. The inhalation flow rate was 20 L/min. The charge levels considered included high charge (MDI), saturation charge (DPI), Boltzmann charge, and neutral. The electrostatic effect manifested itself very differently in deposition pattern. While it was almost unperceivable for ultrafine aerosols (0.04 µm), the effect increased with increasing particle size and could be significant for micron particles (Figure 4). In the case of 10 µm, the surface deposition of high charge (MDI) aerosols was dramatically different from those of the other three charge levels, with amplified particle accumulation in the nasal valve region. Inhalation flow rates of 10 and 30 L/min were also considered. For a given particle charge level, relatively similar deposition patterns were observed among the three flow rates, indicating a relatively small impact of inhalation flow rates under normal breathing conditions.

**Figure 5:**
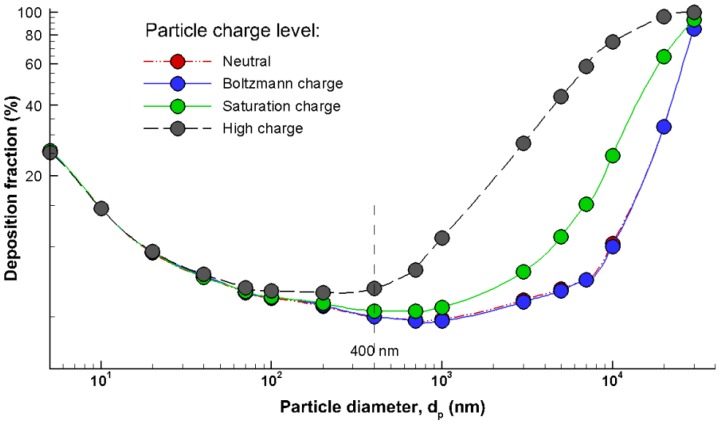
Comparison of deposition fraction as a function of particle size under different charge levels.

Comparisons of total depositions among different charge levels were illustrated in Figure 5 for particles ranging from 5 nm to 30 μm. Inclusion of electric charge was found to increase the overall deposition fraction for particles larger than 40 nm. For particles of 40 nm and smaller, almost no variation was observed among the four charge levels due to their small amount of charges. For particles ranging from 0.4 μm to 10 μm, nasal deposition of the high charge (MDI) particles was approximately one order of magnitude higher than that of neutral particles, and was about two times that of saturation-charged particles. Particles of 30 µm and larger were generally filtered out before the nasal valve (Figure 5) no matter what the particle charge level is. Another prominent feature associated with the charge effect was that Bolzmann charge exerted a negligible effect on total deposition for all particle size considered, which agreed with the finding in Fry [6].

**Figure 6:**
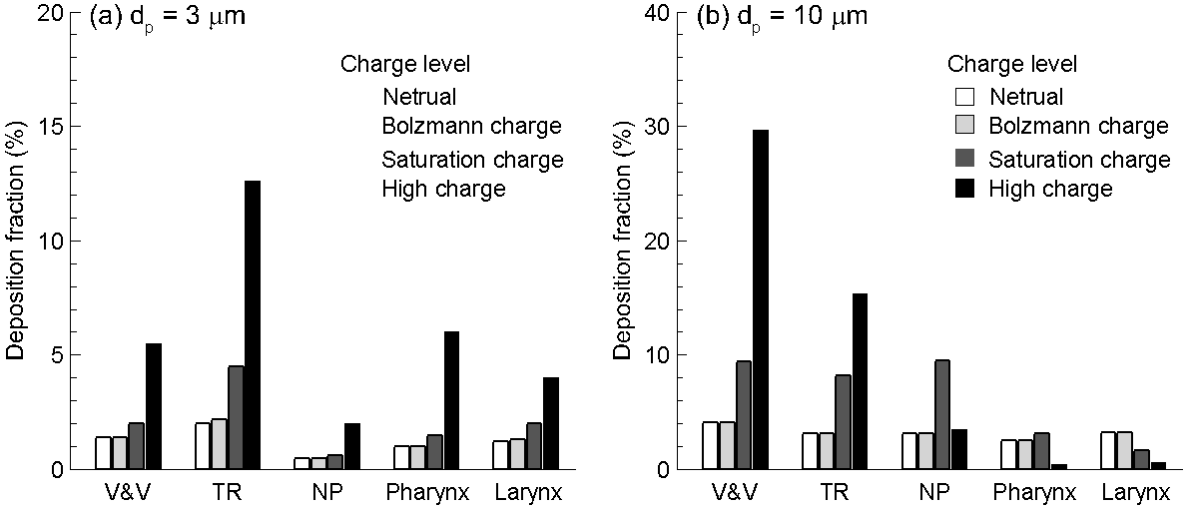
Sub-regional deposition of monodisperse aerosols with different charge levels for particle size of (**a**) 3 µm, and (**b**) 10 µm. For 10 µm particles, deposition shifted to front of the nasal cavity as charge level increased.

The electrostatic effect on nasal deposition was further illustrated in Figure 6 in terms of deposition within specific sections of the nose. The extent of each section was depicted in Figure 1a, and the particle sizes considered were 3 and 10 µm. The deposition variation associated with electrostatic effect was clearly illustrated in this example. Again, no discernible difference was found between particles with neutral and Boltzmann charge for all sections and both particle sizes considered. For 3 µm particles, which are typical of pharmaceutical aerosols, saturation charge enhanced deposition rate in the turbinate region by a factor of two in comparison to neutral or Bolzmann charges (Figure 6a); however, the deposition enhancement on other sub-regions is much smaller. In contrast, high-charge level enhances deposition rates of 3 µm particles in all sub-regions considered, especially in the turbinate region (Figure 6a). Considering 10 µm particles, deposition shifted to the front of the nasal cavity as the charge level increased (Figure 6b). In the nasal vestibule and valve (V & V) region, the deposition fraction with high charge (MDI) and saturation charge (DPI) was about 7.3 and 2.0 times of no-charge deposition, respectively. The deposition enhancement was more pronounced in the turbinate region (TR) relative to the upstream V & V region; however, the MDI and DPI depositions hereof were still 4.3 and 2.4 times that of no-charge particles, respectively (Figure 6b). For nasal sprays that often targeted the capillary-rich mucosa in the turbinate region, this enhancement processed a great potential to improve the efficiency of intranasal drug deliveries. Interestingly, the high-charge deposition was minimal among the four charge levels in the downstream sections such as nasopharynx (NP), pharynx, and larynx. This phenomenon was possibly attributed to the effective filtering in the upstream nasal valve-vestibule (V & V) and turbinate regions. Based on the results of this study, electric charges could substantially alter the nasal dosages from a MDI (high charge) and a neutralizer was recommended to decrease dosage variability. On the other hand, by selecting appropriate electric charges, it was possible to increase depositions in certain site and diminish them in other sites, therefore maximizing the therapeutic outcome and minimizing side effects.

## 4. Conclusions

In this study, electrostatic effects on the deposition of nasally inhaled pharmaceutical aerosols were systemically assessed using an image-based computational modeling approach. Specific findings included:

1. For submicron aerosols, electrostatic charge exerted a discernible but not significant effect on both total and sub-regional depositions;
2. For micrometer particles, which could acquire high levels of electrostatic charge, both total and local deposition values could be altered substantially depending on the aerosol charge;
3. With increasing charge levels, the high-deposition-region shifted toward the front of the nasal passage;
4. Boltzmann charge had a negligible effect on the nasal deposition of both submicron and micrometer aerosols.
